# Does ethnic density influence community participation in mass participation physical activity events? The case of parkrun in England

**DOI:** 10.12688/wellcomeopenres.15657.2

**Published:** 2020-06-18

**Authors:** Robert Smith, Paul Schneider, Alice Bullas, Steve Haake, Helen Quirk, Rami Cosulich, Elizabeth Goyder

**Affiliations:** 1 School of Health and Related Research, University of Sheffield, Regents Court, Sheffield, S1 4DA, UK; 2 Advanced Wellbeing Research Centre, Sheffield Hallam University, Olympic Legacy Park, Sheffield, S9 3TU, UK

**Keywords:** parkrun, Physical Activity, Ethnic Density, Deprivation

## Abstract

**Background:** parkrun has been successful in encouraging people in England to participate in their weekly 5km running and walking events. However, there is substantial heterogeneity in parkrun participation across different communities in England: after controlling for travel distances, deprived communities have significantly lower participation rates.

**Methods:** This paper expands on previous findings by investigating disparities in parkrun participation by ethnic density. We combined geo-spatial data available through the Office for National Statistics with participation data provided by parkrun, and fitted multivariable Poisson regression models to study the effect of ethnic density on participation rates at the Lower layer Super Output Level.

**Results:** We find that areas with higher ethnic density have lower participation rates. This effect is independent of deprivation.

**Conclusions:** An opportunity exists for parkrun to engage with these communities and reduce potential barriers to participation.

## Introduction

parkrun is a collection of free mass participation 5km running events that takes place every Saturday morning. There are currently over 500 locations in England, with a combined weekly attendance of over 100,000. parkrun has been identified as being successful at engaging with individuals who may not otherwise have taken part in organised physical activity ^1, 2^, and there is some evidence that it has increased overall physical activity levels in participants ^3^. Overall, there is a consensus that parkrun has huge public health potential ^4^.

However, qualitative research in Sheffield ^5^ and other areas of the United Kingdom ^6^ identified that parkruns located in more deprived areas have lower attendances, and that ethnic diversity in parkrun was limited. This leads to concern that as with many public health interventions, parkrun is "likely to be responsible for significant intervention generated inequalities in uptake of opportunities for physically active recreation" ^5^.

Undertaking quantitative analysis of the determinants of participation in parkrun is therefore long overdue. Apart from a single previous study from Australia ^7^, with substantial limitations including, as noted by the authors, that "The sample was limited to a non-random sample of parkrun participants in one State of Australia and may not be generalizable to other parkrun populations." (p.21), no other studies have attempted to identify the determinants of participation in parkrun.

Our previous work revealed that there is substantial heterogeneity in parkrun participation across different communities in England: after controlling for geographical distance to nearest event, deprived communities have significantly lower participation rates ^8^. The analysis was able to quantify, for the first time, how participation in parkrun varied in different communities in England. However, the analysis only explored the relationship between participation, access and deprivation and did not consider ethnic density as a potential determinant of participation in parkrun. Evidence from survey data shows that non-White-British individuals in England are less likely to be physically active, and to engage in sport in general ^9^. We thus hypothesised that at the community level, areas with higher ethnic density have lower levels of participation in parkrun.

## Methods

### Ethical statement

Ethical approval was obtained from the Sheffield Hallam University Ethics Committee (ER10776545). We did not collect any personal information, but only used aggregate secondary data. The parkrun Research Board approved this research project, and three of its members (AMB; EG, SSJH) were actively involved in it.

### Data sources

We undertook an ecological analysis of parkrun participation in England in 2018. Data was obtained from multiple sources (see Table 1) for the 32,844 Lower layer Super Output Areas (LSOAs) in England, each of which is a geographical area containing around 1,500 people. parkrunUK provided data on the number of parkrun finishers from each LSOA in England between the 1st January and 10th December 2018, which we use as a proxy for parkrun participation, although we appreciate that people participate in parkrun in other ways (e.g. volunteering). We also used parkrun event location data, which are publicly available on the parkrunUK website.

The rest of the data, including Index of Multiple Deprivation (IMD) Score, Ethnic Density, Rural-Urban Classification, Population Density, Percentage Working Age and LSOA centroids were obtained from the Office of National Statistics (ONS). Descriptions of variables and sources are listed in Table 1, and all data is provided open source as *Underlying data* and on the author’s GitHub page ( https://github.com/bitowaqr/DoPE) ^10^.

**Table 1.** Variables used in the analysis.

Variable	Description	Source
Finishers	Number of parkrun finishers during period	parkrunUK (2018)
IMD score	Index of Multiple Deprivation score	ONS (2019)
Population	Total number of inhabitants	ONS (2019)
Pop density	Population density (pop */*km ^2^)	ONS (2019)
Rural-urban classification	Rural-urban classification (binary)	ONS (2019)
Ethnic density	Proxy: Percentage of population non-White-British	ONS (2019)
Distance	Distance from LSOA centroid to nearest parkrun	derived
Non-working-age	Percent of population not 16-65	ONS (2019)
Participation rate	Number of finishes */*1000 population	derived

IMD, Index of Multiple Deprivation; LSOA, Lower layer Super Output Area; ONS, Office for National Statistics.

### Data analysis

The merged data-set contains complete data for all LSOAs, and therefore all LSOA were included within the analysis, which was conducted using R software environment version 3.5.1 (2018-07-02) ^11^. We first used a simple colour plot to display the relationship between deprivation, ethnic density and parkrun participation graphically using ggplot ^12^. We then used Poisson regression models, commonly used when working with count data, to estimate the relationship between ethnic density, deprivation and parkrun participation, controlling for potential confounding variables including: population density, population, age and distance to nearest parkrun event.

## Results

### Descriptive statistics

Descriptive statistics are shown in Table 2. Participation in parkrun varies across LSOAs, with around half of all communities (LSOA) averaging less than one finisher per week per 1,000 people. Approximately a quarter average between one and two finishers, and around an eighth between two and three finishers. There is considerable variation in ethnic density, with most LSOAs having a large majority of White-British residents, and few areas having over 50% non-White-British residents. Deprivation score is positively skewed, meaning that most areas have low deprivation, with a few very deprived areas. Finally, around 70% of LSOAs are within 5km, the parkrun distance, of a parkrun. Again, this is positively skewed with half of all LSAOs being within 3.5km of their nearest event.

There is a negative correlation between participation and the following: deprivation (IMD), distance to nearest parkrun, population density and ethnic density. Ethnic density is strongly positively correlated with population density, negatively correlated with percentage non working age, and moderately positively correlated with IMD, suggesting that areas with higher ethnic density are more densely populated overall, more deprived and have a higher percentage of working age people.

**Figure 1.**
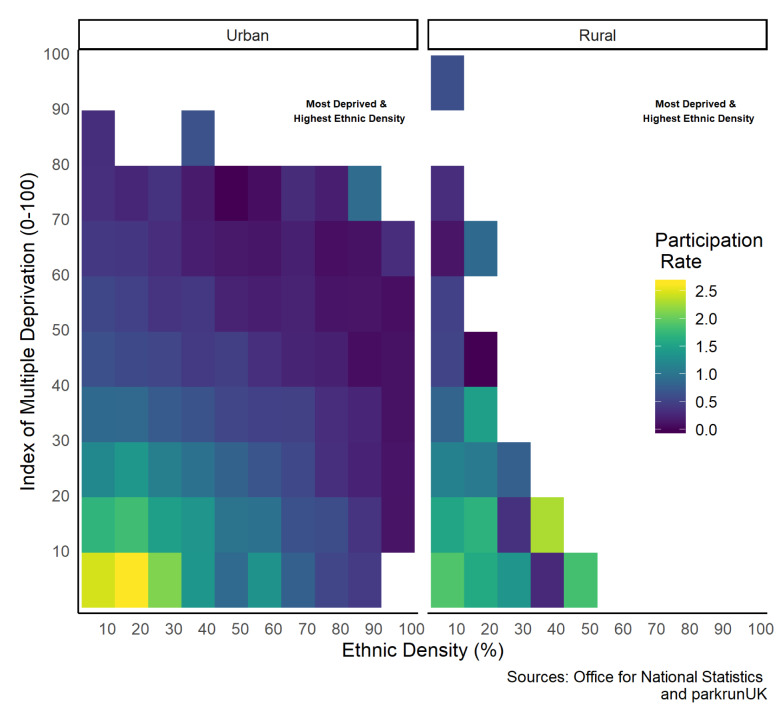
Colour plot for parkrun participation by rural-urban classification, Index of Multiple Deprivation and ethnic density.

The colour plots in Figure 1 show the participation rates for LSOA by deprivation and ethnic density for urban and rural areas ^13^. Yellow, green and blue indicate high, moderate and low levels of participation respectively. The plot shows that participation is generally greatest in areas that have low levels of deprivation and low levels of ethnic density (bottom left), and lowest in areas with high levels of deprivation and high ethnic density (top-right). Areas with either high deprivation, or high ethnic density, tended to have low participation, suggesting that both are important independently. The relationship was robust to urban major areas and urban minor areas but did not hold in rural areas where data was more limited. It is important to note that we do not control for other factors, such as the age of residents or the population density, which are known confounders of this relationship.

**Table 2.** Descriptive statistics.

Statistic	N	Mean	St. Dev.	Min	Pctl(25)	Median	Pctl(75)	Max
Finishers	32,844	123.6	128.9	0	33	86	172	1,659
IMD score	32,844	21.7	15.3	0.5	9.9	17.6	29.6	92.7
Ethnic density (%)	32,844	13.8	18.7	0.0	2.3	5.2	16.7	99.3
Distance (km)	32,844	4.7	4.3	0.04	2.0	3.5	6.0	76.4
Population	32,844	1,666.3	363.6	523	1,446	1,598	1,800	9,551
Pop density (pop */*km ^2^)	32,844	4,423.7	4,506.0	2.5	1,266.8	3,523.7	5,865.3	103,400.0
Non-working-age (%)	32,844	42.6	7.9	1.2	38.9	43.2	47.4	73.6
Participation rate	32,844	1.4	1.5	0.0	0.4	1.0	2.0	15.6

IMD, Index of Multiple Deprivation.

### Poisson model

The results of three Poisson regression models are shown in Table 3. All models include the control variables: population density, distance to nearest event and percentage of the population of non-working age. Model 1 includes IMD Score, Model 2 includes ethnic density and Model 3 includes both IMD and ethnic density. All coefficients are significant at the p<0.01 level.

Model 1 shows that, controlling for population density, distance to nearest event and age of population, areas with higher IMD (more deprived) have lower participation.

Model 2 shows that, with the same controls, areas with higher ethnic density have lower participation.

Model 3 shows that when both independent variables (IMD and ethnic density) are included their coefficients decrease, suggesting that some of the effect previously attributed to deprivation is indeed due to lower participation in areas with higher ethnic density.

**Table 3.** Poisson log-link generalised linear model results.

	Dependent variable: Finishers		
	Model 1 (IMD)	Model 2 (Ethnic density)	Model 3 (IMD and ethnic density)
IMD score	−0.037 * (0.00005)		−0.034 * (0.00005)
Ethnic density (%)		−.020 * (0.00004)	−0.052 * (0.00004)
Pop density (pop */*km ^2^)	−0.107 * (0.0004)	−0.118 * (0.0004)	−0.070 * (0.0004)
Distance (km)	−0.107 * (0.0002)	−0.116 * (0.0002)	−0.112 * (0.0002)
Non-working-age (%)	0.006 * (0.00007)	0.002 * (0.00007)	−0.001 * (0.00007)
Constant	−0.913 * (0.005)	−1.068 * (0.005)	−0.737 * (0.005)
Observations	32,844	32,844	32,844
Log Likelihood	−1,301,151.000	−1,554,894.000	−1,231,308.000
Akaike Inf. Crit.	2,602,312.000	3,109,799.000	2,462,628.000

Note: Std. Error in parenthesis              *p *<*0.01 IMD, Index of Multiple Deprivation.

## Discussion

Our findings show that more deprived areas and areas with higher ethnic density have lower participation rates. This effect persists after controlling for other area characteristics such as deprivation, access to events and population density. While our previous analysis ^8^ showed that participation in parkrun is lower in more deprived communities, the present results suggest that a small part of the negative effect on participation previously attributed to deprivation can actually be attributed to ethnic density. parkrun’s vision of creating a “healthier and happier planet by continually breaking down barriers to participation and bringing people together from all walks of life whenever they want to come along” (p.5) ^14^ has potential to improve both population physical activity and community engagement. Identifying the determinants of participation at the community level is a useful first step, but qualitative work to understand why and how these determinants influence participation is an obvious next step. Replicating this study in several years will enable parkrun to monitor trends in participation from different groups in society, and therefore the effectiveness of efforts to reach minority communities and those living in deprived areas.

### Limitations

This analysis is ecological and therefore it is not possible to make conclusions at an individual level without risking an ecological inference fallacy. We have been careful throughout to make conclusions at the level of the LSOA, rather than the individual. Nevertheless, given that the evidence at the individual level points to lower participation in organised sport by those from ethnic minority backgrounds ^9^, we think it is likely that the same effect exists at the individual level.

Our dependent variable is the number of finishers by residents of each LSOA. This is a count variable where each walk or run finished is treated equally (e.g. 10 finishes by one person is equal to 10 people completing one event). We cannot draw inferences on the number of people who took part within each LSOA at some point in the year, but instead focus on the total finisher count. We do not expect that this will affect the core finding of the paper.

We use percent non-White-British as a crude proxy for ethnic density, and do not estimate participation by ethnic groups separately. It is possible that there are significant differences between participation rates of different minority ethnic groups. Future analysis could look into which groups are more or less engaged in order to better understand the underlying causes of participation. Furthermore, we controlled for several variables that we thought would influence participation but it is possible that there are other confounding factors that have not been included.

## Conclusions

parkrun is already in the process of increasing the number of events in deprived areas of England to encourage participation from disadvantaged groups. Our findings show, however, that in addition to deprivation and access, ethnic density is another important determinant of participation. Breaking down barriers to engagement in parkrun has the potential to improve overall population physical activity and therefore improve overall health and reduce health inequalities.

## Data availability

### Underlying data

Zenodo: RobertASmith/DoPE_Public: Determinants of parkrun Engagement v1.0. https://doi.org/10.5281/zenodo.3596841 ^10^

This project contains the following underlying data:

- /output (folder contains the cleaned data file in CSV format)
- /raw_data/England_lsoa_2011_centroids (LSOA centroid data in DBF, PRJ, SHP and SHX formats)
- /raw_data/IoD2019_Population_Denominators.csv (Non-working age data)
- /raw_data/IoD2019_Scores.csv (Index of Multiple Deprivation score data)
- /raw_data/LSOA_Ethnicity.csv (Ethnic density data)
- /raw_data/LSOA_Rural_Urban_Classification_2011.csv (Rural-urban classification data)
- /raw_data/Mid-2017 Population Density.csv (Population density data)
- /raw_data/parkrun_data (location data and number of parkrun finishers in CSV format)

Data are available under the terms of the Creative Commons Zero “No rights reserved” data waiver (CC0 1.0 Public domain dedication).

## Software availability

Source code available from: https://github.com/ bitowaqr/DoPE

Archived source code at time of publication: https://doi.org/10.5281/zenodo.3596841 ^10^

License: MIT
