# Supplementary material for: Does ethnic density influence community participation in mass participation physical activity events? The case of parkrun in England
Source: Wellcome Open Res. 2020 Jun 18;5:9. Originally published 2020 Jan 16. [Version 2] doi: 10.12688/wellcomeopenres.15657.2 (PMC7065574; doi:10.12688/wellcomeopenres.15657.2)
Supplement: Peer review history

# Peer review: Reviewer response for version 2

**DOI:** 10.21956/wellcomeopenres.17628.r39168

**Published:** 2020-06-29

**Authors:** Stephen Senn

**Affiliations:** 1 Consultant Statistician, Edinburgh, UK

I shall approve this revision. Nevertheless, I think that the authors' response to my criticism of the form of Poisson regression that they used is disappointing. The response boils down to agreeing that the technique used is questionable in general but then claiming that it doesn't matter in this particular instance. This claim is very probably true but just possibly false and it would have been an easy matter for the authors to check: far easier than for any reviewer to do so.

 I am not, except by occasional necessity, an R programmer (my usual package for analysis is Genstat) however, looking at my copy of Michael Crawley's book it seems that all that is necessary is to use ***quasipoisson*** as an alternative argument to ***poisson*** in the ***glm*** function (See p582) ^1^. I am baffled as to why the authors did not think it was worthwhile making the effort to do so.

Is the work clearly and accurately presented and does it cite the current literature?

Yes

If applicable, is the statistical analysis and its interpretation appropriate?

Partly

Are all the source data underlying the results available to ensure full reproducibility?

Yes

Is the study design appropriate and is the work technically sound?

Partly

Are the conclusions drawn adequately supported by the results?

Partly

Are sufficient details of methods and analysis provided to allow replication by others?

Partly

Reviewer Expertise:

Statistical methodology; medical statistics; drug development; clinical trials; epidemiology.

I confirm that I have read this submission and believe that I have an appropriate level of expertise to confirm that it is of an acceptable scientific standard.

## References

[1] : The R book (second edition). *John Wiley*.2013;

# Response

**Published:** 2020-06-29

**Authors:** Robert Smith

**Affiliations:** University of Sheffield, UK

When responding to reviewer 3’s comments on the use of Poisson regression we stated that we did not think that there was any way the alternative method would change the results, but that others could use the open access code and data to test this. We are keen advocates of open science and so hoped that others would do this (in fact they already have at several R-hackathon events).

The reviewer has since responded that this is disappointing, and that we should have re-run the analysis, We agree - simply making code and data publicly available does not equal full transparency, particularly when people are trained in different programming languages. We therefore present the results of models 1, 2 and 3 (link below) using a Poisson (P) and QuasiPoisson (QP) regression model. As suspected, the changes do not affect the findings of the study.

We once again thank Reviewer 3 for their useful comments, and apologize for not re-running this relatively simple change to the analysis in the first instance.

https://github.com/ScHARR-PHEDS/DoPE_Public/blob/master/report/QuasiPoisson.pdf

# Peer review: Reviewer response for version 2

**DOI:** 10.21956/wellcomeopenres.17628.r39166

**Published:** 2020-06-19

**Authors:** Anne Grunseit

**Affiliations:** 1 The Australian Prevention Partnership Centre, NSW, Australia; 2 Sydney School of Public Health, University of Sydney, Sydney, Australia

No further comments.

Is the work clearly and accurately presented and does it cite the current literature?

Yes

If applicable, is the statistical analysis and its interpretation appropriate?

Yes

Are all the source data underlying the results available to ensure full reproducibility?

Yes

Is the study design appropriate and is the work technically sound?

Yes

Are the conclusions drawn adequately supported by the results?

Yes

Are sufficient details of methods and analysis provided to allow replication by others?

Partly

Reviewer Expertise:

Social epidemiology and evaluation in the prevention of lifestyle-related chronic disease.

I confirm that I have read this submission and believe that I have an appropriate level of expertise to confirm that it is of an acceptable scientific standard.

# Peer review: Reviewer response for version 2

**DOI:** 10.21956/wellcomeopenres.17628.r39167

**Published:** 2020-06-18

**Authors:** Gozde Ozakinci

**Affiliations:** 1 School of Medicine, University of St Andrews, St Andrews, UK

The authors have addressed my query.

Is the work clearly and accurately presented and does it cite the current literature?

Yes

If applicable, is the statistical analysis and its interpretation appropriate?

I cannot comment. A qualified statistician is required.

Are all the source data underlying the results available to ensure full reproducibility?

Yes

Is the study design appropriate and is the work technically sound?

Yes

Are the conclusions drawn adequately supported by the results?

Yes

Are sufficient details of methods and analysis provided to allow replication by others?

Yes

Reviewer Expertise:

Health psychology; community physical activity participation.

I confirm that I have read this submission and believe that I have an appropriate level of expertise to confirm that it is of an acceptable scientific standard.

# Peer review: Reviewer response for version 1

**DOI:** 10.21956/wellcomeopenres.17155.r38442

**Published:** 2020-04-30

**Authors:** Stephen Senn

**Affiliations:** 1 Consultant Statistician, Edinburgh, UK

The results of this interesting study are nicely presented and generally well discussed. There are three aspects of the statistical analysis that may be criticised.

 First, the authors have used Poisson regression, pointing out that this is commonly used for count data. However, the validity of a Poisson model relies on the assumption that bedrock variability has been reached and this in turn requires that a complete and correct model incorporating all relevant factors has been employed. Furthermore, the Poisson model is a single parameter model with variance equal to expectation. This means that, unlike the Normal model, there is no further play in the model to allow for hidden covariates. This is usually dealt with in one of two ways by modellers. The first is to incorporate a hidden 'frailty' or 'proneness' parameter. If this is assumed to follow a gamma distribution, then, integrating this out leads to a negative binomial model. This is a two parameter model that can thus allow variances to be greater than predicted by expectation. The second is to check the residual deviance and compare this to the degrees of freedom. The ratio of one to the other then gives a factor by which variances of estimates should be inflated to allow for lack of fit due to hidden random factors. I found no discussion of this point in the paper so can only assume that simple Poisson regression was used, in which case it is likely that the quoted standard errors are too small (See Senn ^1^ p13 for a discussion).

 The second point is that population should perhaps have been used as an offset in the model (see McCullagh and Nelder ^2^ p206). Opinions might differ as to how appropriate this is but I would have expected to see it discussed.

 The third point is that in controlling for measures of deprivation the authors are asking the question 'given equal deprivation is ethnicity predictive of participation?'. This, is a partial "effect". It may underestimate the role of ethnicity since part of this may be via a tendency to suffer greater deprivation. I am not suggesting that the authors' chosen analysis is inappropriate in controlling for these factors; I am just suggesting that it merits discussion.

Is the work clearly and accurately presented and does it cite the current literature?

Yes

If applicable, is the statistical analysis and its interpretation appropriate?

Partly

Are all the source data underlying the results available to ensure full reproducibility?

Yes

Is the study design appropriate and is the work technically sound?

Partly

Are the conclusions drawn adequately supported by the results?

Partly

Are sufficient details of methods and analysis provided to allow replication by others?

Partly

Reviewer Expertise:

Statistical methodology; medical statistics; drug development; clinical trials; epidemiology.

I confirm that I have read this submission and believe that I have an appropriate level of expertise to confirm that it is of an acceptable scientific standard, however I have significant reservations, as outlined above.

## References

[1] : Modelling in drug development. *Wiley*.2011;35-49

[2] : Generalized Linear Models (Second ed.). *Chapman and Hall*.1989;

# Response

**Published:** 2020-06-05

**Authors:** Robert Smith

**Affiliations:** University of Sheffield, UK

Thank-you very much for reviewing this paper. It is fantastic that we have been able to get peer review on this paper in such a short period of time, so that the findings can have immediate impact. 

Our responses to your three points are below: 

1) On the validity of a poisson regression model in this instance. 

We used a simple Poisson regression model and acknowledge that the standard errors around coefficient point estimates might be underestimated. However, with a sample size of more than 32,000 LSOAs, this problem seems theoretical, as the standard errors are very small. In model 3, for example, the point estimate for ‘ethnic density’ is –0.052, and the respective standard error is 0.00004. To us it seems unlikely that using a negative binomial model would have any relevant effect on the parameter uncertainty and/or the interpretation of the results. However, that being said, we would be very pleased if somebody who is interested would like to investigate this further. All data and code used in this paper are here: https://doi.org/10.5281/zenodo.3596841.

2) On population offset:

Thank you for pointing this out – we did indeed use population as an offset variable in the Poisson model, but failed to report this properly in the method section. The respective paragraph has been revised in the following way:

*We then used Poisson regression models, commonly used when working with count data, to estimate the relationship between ethnic density, deprivation and parkrun participation, controlling for potential confounding variables including: population density, (population,) age and distance to nearest parkrun event. The LSOA’s total population was used as an offset variable.*

() = removed.

3) On the partial effect issue: 

This is an important point: deprivation might be endogenous, i.e. on the LSOA level, there might be a ‘flow of causality’ from ethnic density to the level of deprivation. In this case, the effect of ethnic density on participation would be underestimated, as the effect would be partly (and falsely) attributed to deprivation. While controlling for this effect in the statistical model would be a challenge with the data we have, we fully agree with the reviewer that this point deserve mentioning, and thank him for his thoughtful suggestion. The following sentence was added to the discussion:

*Finally, it can be assumed that there are some causal relationships between the predictors in our model (e.g. between percentage working age or ethnic density and Deprivation). Future studies should consider conducting mediation analysis, to further disentangle their direct and indirect effects.*

We now have participation data for every year from 2010-2019. While it is unlikely that we will be able to solve this specific issue (ethnic density and deprivation don't vary much year by year) we hope to better understand the determinants of parkrun using this more detailed data.

# Peer review: Reviewer response for version 1

**DOI:** 10.21956/wellcomeopenres.17155.r37796

**Published:** 2020-03-09

**Authors:** Gozde Ozakinci

**Affiliations:** 1 School of Medicine, University of St Andrews, St Andrews, UK

This is a much needed analysis of potential differences in participation in parkrun. parkrun's popularity raises questions about health inequalities and these sophisticated analyses help us examine the different role that ethnic density and deprivation may play.

 I can't comment on the statistical analyses as they are beyond my expertise. The focus of the analyses is the total count of finishers. However, it's not clear to me why a more 'nuanced' approach has not been possible - for instance is it possible to analyse the data in a way that shows the impact of 'parkrun tourism'. Is it possible that this inflates the numbers in some way? By the way, I don't think this will change the conclusions but just pointing it out to encourage authors to give more rationale.

Is the work clearly and accurately presented and does it cite the current literature?

Yes

If applicable, is the statistical analysis and its interpretation appropriate?

I cannot comment. A qualified statistician is required.

Are all the source data underlying the results available to ensure full reproducibility?

Yes

Is the study design appropriate and is the work technically sound?

Yes

Are the conclusions drawn adequately supported by the results?

Yes

Are sufficient details of methods and analysis provided to allow replication by others?

Yes

Reviewer Expertise:

Health psychology; community physical activity participation.

I confirm that I have read this submission and believe that I have an appropriate level of expertise to confirm that it is of an acceptable scientific standard.

# Response

**Published:** 2020-05-04

**Authors:** Robert Smith

**Affiliations:** University of Sheffield, UK

**Thank-you for the nice comments on the paper. We appreciate you taking the time to review. To answer your question: The number of finishers is aggregated by geographic area in which the finisher lives, therefore parkrun tourism should not influence the results.**

**For example: if I live in an affluent area and travel to a deprived area to do a parkrun this counts towards runs done in affluent areas, not in deprived areas. So, I think you are correct in saying that it would not change the conclusions.**

**However, what would be interesting in this case is to understand why relatively local parkrun tourism occurs (do people not go to their nearest parkrun because another is more pleasant).**

# Peer review: Reviewer response for version 1

**DOI:** 10.21956/wellcomeopenres.17155.r37608

**Published:** 2020-03-09

**Authors:** Anne Grunseit

**Affiliations:** 1 The Australian Prevention Partnership Centre, NSW, Australia; 2 Sydney School of Public Health, University of Sydney, Sydney, Australia

Comments

 This paper describes an ecological study of the reach of parkrun in terms of ethnic diversity in parkrun. The paper addresses one of the outstanding issues in the evidence base on this physical activity phenomenon and uses a novel (to parkrun research) method to investigate it. The paper is essentially well-written, has interesting and useful findings and has a sound methodological approach. The question of the effect of clustering within the definition of the participation measure is my only substantive concern, and just requires explanation (see below). Otherwise I have a number of minor comments which should be considered before accepting this article for indexing.

*Introduction*

 Par 1, line 1: I don’t think “collection” is the right word – it implies that they are in one place when parkrun’s main asset is that it is disseminated. A small wording revision should address this.

 Par 4, last sentence: word missing: density would have…

  It might be helpful to have a line about physical activity rates among ethnically diverse populations here – sport is not the only form of physical activity and therefore you need to demonstrate that total activity, which is what matters for health, is also lower than for other groups/communities.

*Methods*

 Under data sources, could the authors please indicate whether “finishers” were unique or just a total count ignoring repeat participation (this is mentioned only in the limitations but should be earlier). The authors should describe what the potential implications are of this for the analysis and interpretation – they do mention that they do not expect it change the results in the limitations but do not provide the rationale for such a conclusion. It also begs the question why they did not use unique persons because it would be possible to do this with parkrun data. In other words they should explain why they chose to operationalise participation this way.

 Percentage working age – is there any reason why the authors chose this particular variable for age and how does it relate to the objectives of the analysis?

 LSOAs - Could the authors describe why this particular level of spatial classification was used – parkruns draw on varying areas depending on population density but also the proximity to other parkruns. Could they also state whether there were any cases where two or more SOAs were equidistant and if so how were they allocated.

 Data analysis – the authors should describe what assumptions for poisson regression were tested (over-dispersion for example). They should also describe how age was operationalised.

*Results*

 The authors talk about ethnic diversity but do not give the reader much idea about what ethnicities this covers in these areas. The authors should describe this somewhere (intro, methods, results) to give the non-UK reader some further context.

 Par 1: third last line: Remove “the parkrun distance” as it is not relevant as such to the point being made.

 Par 2: The result for age has been reversed in the results from how it was described in the methods which actually makes it more difficult to understand. Is there any reason why you talk about % non-working age rather than % working age? At the very least it should be consistent between methods and results.

 Par 3: you make reference to major areas and urban minor areas but have not defined this anywhere. Either here or in the methods would be suitable.

 Par on Model 3: As I read the table, despite the attenuation of the effects for IMD and Ethnic density they remained significant in the model – should be explicitly stated in the text.

*Limitations*

 The authors should also note that this research was conducted in one country and the associations may be different in other countries with different geo-demographic patterns and parkrun density.

Is the work clearly and accurately presented and does it cite the current literature?

Yes

If applicable, is the statistical analysis and its interpretation appropriate?

Yes

Are all the source data underlying the results available to ensure full reproducibility?

Yes

Is the study design appropriate and is the work technically sound?

Yes

Are the conclusions drawn adequately supported by the results?

Yes

Are sufficient details of methods and analysis provided to allow replication by others?

Partly

Reviewer Expertise:

Social epidemiology and evaluation in the prevention of lifestyle-related chronic disease.

I confirm that I have read this submission and believe that I have an appropriate level of expertise to confirm that it is of an acceptable scientific standard.

# Response

**Published:** 2020-05-04

**Authors:** Robert Smith

**Affiliations:** University of Sheffield, UK

Thank-you for taking the time to review and for the many useful comments (which I overwhelmingly agree with).

*Introduction*

Par 1, line 1: I don’t think “collection” is the right word – it implies that they are in one place when parkrun’s main asset is that it is disseminated. A small wording revision should address this.

**RS: Agreed - changed to use network!**

Par 4, last sentence: word missing: density would have…

**RS: Happy to change this.**

 It might be helpful to have a line about physical activity rates among ethnically diverse populations here – sport is not the only form of physical activity and therefore you need to demonstrate that total activity, which is what matters for health, is also lower than for other groups/communities.

**RS: We state that “non-White-British individuals in England are less likely to be physically active”.**

*Methods*

Under data sources, could the authors please indicate whether “finishers” were unique or just a total count ignoring repeat participation (this is mentioned only in the limitations but should be earlier).

**RS: Agreed, this is important for clarity and we have updated this. The number is the total number of finishers (the data we are provided by parkrun  is aggregated number of runs by area in order to maintain anonymity). It would be really nice to do with individual level data - but then we would not be able to make the data open access.**

The authors should describe what the potential implications are of this for the analysis and interpretation – they do mention that they do not expect it change the results in the limitations but do not provide the rationale for such a conclusion. It also begs the question why they did not use unique persons because it would be possible to do this with parkrun data. In other words they should explain why they chose to operationalise participation this way.

**RS: Agreed, we have updated the publication to state why it is not possible to do this with the data we have, and also that this limitation may causes biases in the results.**

Percentage working age – is there any reason why the authors chose this particular variable for age and how does it relate to the objectives of the analysis?

**RS: This variable is available in the ONS dataset and was used to control for the effect of having care homes in one area. We wanted to try and control for the effect of having a large community of particularly old people (e.g. a care home or nursing facility) in one LSOA.**

LSOAs - Could the authors describe why this particular level of spatial classification was used – parkruns draw on varying areas depending on population density but also the proximity to other parkruns. Could they also state whether there were any cases where two or more SOAs were equidistant and if so how were they allocated.

**RS: The LSOA is commonly used as it is the most detailed level of statistic available from the ONS. There were no cases where a LSOA centroid was equidistant.**

Data analysis – the authors should describe what assumptions for poisson regression were tested (over-dispersion for example). They should also describe how age was operationalised.

**RS: We are in the process of responding to reviewer 3 whose main focus was on these issues. Age was operationalised as a simple percentage non-working age.**

*Results*

The authors talk about ethnic diversity but do not give the reader much idea about what ethnicities this covers in these areas. The authors should describe this somewhere (intro, methods, results) to give the non-UK reader some further context.

**RS: This is defined as the percentage of the population who are non-white-British. We didn't look into specific ethnicity, although it would be possible using the data from the ONS.**

Par 1: third last line: Remove “the parkrun distance” as it is not relevant as such to the point being made.

**RS: Agreed, made change.**

Par 2: The result for age has been reversed in the results from how it was described in the methods which actually makes it more difficult to understand. Is there any reason why you talk about % non-working age rather than % working age? At the very least it should be consistent between methods and results.

**RS: This is actually a typo in data sources (we have now changed this to match).**

Par 3: you make reference to major areas and urban minor areas but have not defined this anywhere. Either here or in the methods would be suitable.

**RS: We have updated the data sources to include a description of rural-urban classification.**

Par on Model 3: As I read the table, despite the attenuation of the effects for IMD and Ethnic density they remained significant in the model – should be explicitly stated in the text.

**RS: Agreed, updated.**

*Limitations*

The authors should also note that this research was conducted in one country and the associations may be different in other countries with different geo-demographic patterns and parkrun density.

**RS: Agreed, this point have now been added, along with a call to replicate in other countries. We are particularly keen to see this work replicated and so have made all data and code open access - researchers in other countries with access to that country's ONS equivalent data could easily replicate this work.**
